# Supplementary figures and images for: Performance of five dynamic models in predicting tuberculosis incidence in three prisons in Thailand
Source: PLoS One. 2025 Jan 24;20(1):e0318089. doi: 10.1371/journal.pone.0318089 (PMC11761622; doi:10.1371/journal.pone.0318089)

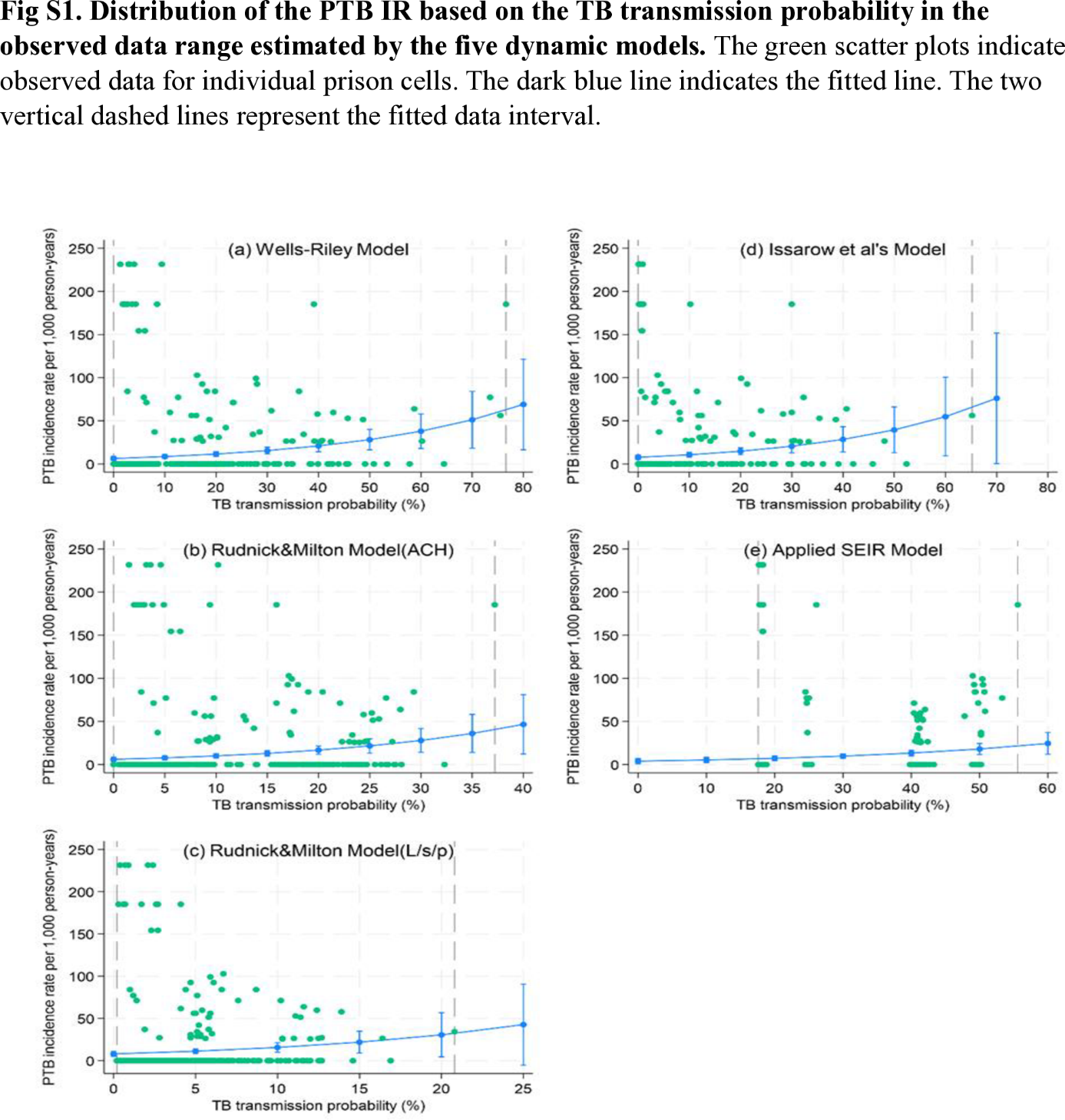

Supplement: S1 Fig — The green scatter plots indicate observed data for individual prison cells. The dark blue line indicates the fitted line. The two vertical dashed lines represent the observed data interval. (DOCX) [file pone.0318089.s001.docx]
