# Supplementary material for: Performance of five dynamic models in predicting tuberculosis incidence in three prisons in Thailand
Source: PLoS One. 2025 Jan 24;20(1):e0318089. doi: 10.1371/journal.pone.0318089 (PMC11761622; doi:10.1371/journal.pone.0318089)
Supplement: S1 Table — (DOCX) [file pone.0318089.s002.docx]

**S1 Table** Akaike information criterion (AIC) and the Bayesian information criterion (BIC) values of the examined count models in determining the association of baseline TB transmission probability with the PTB incidence.

| **Count Model** | **AIC** | **BIC** |
| --- | --- | --- |
| ***Classic Wells–Riley model*** | | |
| Poisson Regression Model (PRM) | 1076.437 | 1086.223 |
| Negative Binomial Regression Model (NBRM) | 1006.171 | 1020.849 |
| Zero-inflated Poisson regression Model (ZIP) | 1014.252 | 1033.823 |
| Zero-inflated negative binomial (ZINB) regression model | 998.542 | 1023.005 |
| Cragg hurdle regression model (HRM) | 1092.316 | 1116.779 |
| ***Rudnick&Milton(ACH) Model*** | | |
| Poisson Regression Model (PRM) | 1086.250 | 1096.036 |
| Negative Binomial Regression Model (NBRM) | 1007.077 | 1021.755 |
| Zero-inflated Poisson regression Model (ZIP) | 1012.892 | 1032.463 |
| Zero-inflated negative binomial (ZINB) regression model | 1005.345 | 1012.892 |
| Cragg hurdle regression model (HRM) | 1092.300 | 1116.764 |
| ***Rudnick&Milton(L/s/p) Model*** | | |
| Poisson Regression Model (PRM) | 1116.459 | 1126.245 |
| Negative Binomial Regression Model (NBRM) | 1027.060 | 1041.738 |
| Zero-inflated Poisson regression Model (ZIP) | 1018.619 | 1038.189 |
| Zero-inflated negative binomial (ZINB) regression model | 1013.505 | 1037.968 |
| Cragg hurdle regression model (HRM) | 1092.255 | 1116.718 |
| ***Issarow et al Model*** | | |
| Poisson Regression Model (PRM) | 951.366 | 961.152 |
| Negative Binomial Regression Model (NBRM) | 915.317 | 929.995 |
| Zero-inflated Poisson regression Model (ZIP) | 933.813 | 953.384 |
| Zero-inflated negative binomial (ZINB) regression model | 916.899 | 941.362 |
| Cragg hurdle regression model (HRM) | 1073.347 | 1048.883 |
| ***Applied SEIR Model*** | | |
| Poisson Regression Model (PRM) | 1013.465 | 1023.251 |
| Negative Binomial Regression Model (NBRM) | 962.220 | 976.898 |
| Zero-inflated Poisson regression Model (ZIP) | 965.520 | 985.091 |
| Zero-inflated negative binomial (ZINB) regression model | 964.591 | 989.055 |
| Cragg hurdle regression model (HRM) | 1077.503 | 1101.966 |

AIC=Akaike information criterion BIC= Bayesian information criterion
