# Supplementary material for: Performance of five dynamic models in predicting tuberculosis incidence in three prisons in Thailand
Source: PLoS One. 2025 Jan 24;20(1):e0318089. doi: 10.1371/journal.pone.0318089 (PMC11761622; doi:10.1371/journal.pone.0318089)
Supplement: S2 Table — (DOCX) [file pone.0318089.s003.docx]

**S2 Table** Comparison of mean predicted projected probabilities and observed proportions of each count of PTB cases in five dynamic models

| **Wells–Riley Model** | | | | | | | | | |  | **Issarow et al.’s Model** | | | | |
| --- | --- | --- | --- | --- | --- | --- | --- | --- | --- | --- | --- | --- | --- | --- | --- |
|  | |  | | **Observed** | | **Fitted** | |  | |  |  |  | **Observed** | **Fitted** |  |
| **# PTB cases** | | **Abs. Freq.** | | **Rel. Freq.** | | **Rel. Freq.** | | **Abs. Dif.** | |  | **# PTB cases** | **Abs. Freq.** | **Rel. Freq.** | **Rel. Freq.** | **Abs. Dif.** |
| 0 | | 589 | | 0.9034 | | 0.9912 | | 0.0878 | |  | 0 | 589 | 0.9034 | 0.991 | 0.0876 |
| 1 | | 46 | | 0.0706 | | 0.0087 | | 0.0618 | |  | 1 | 46 | 0.0706 | 0.009 | 0.0616 |
| 2 | | 15 | | 0.023 | | 7.90E−05 | | 0.0229 | |  | 2 | 15 | 0.023 | 8.00E−05 | 0.0229 |
| 3 | | 2 | | 0.0031 | | 1.10E−06 | | 0.0031 | |  | 3 | 2 | 0.0031 | 1.00E−06 | 0.0031 |
| 4 | | 0 | | 0 | | 2.50E−08 | | 2.50E−08 | |  | 4 | 0 | 0 | 2.10E−08 | 2.10E−08 |
| 5 | | 0 | | 0 | | 7.10E−10 | | 7.10E−10 | |  | 5 | 0 | 0 | 6.30E−10 | 6.30E−10 |
| 6 | | 0 | | 0 | | 2.20E−11 | | 2.20E−11 | |  | 6 | 0 | 0 | 2.30E−11 | 2.30E−11 |
| 7 | | 0 | | 0 | | 7.10E−13 | | 7.10E−13 | |  | 7 | 0 | 0 | 8.90E−13 | 8.90E−13 |
| 8 or more | | 0 | | 0 | | 9.10E−09 | | 9.10E−09 | |  | 8 or more | 0 | 0 | 5.10E−09 | 5.10E−09 |
| Sum | | 652 | | 1.0001 | | 0.99998 | | 0.1756 | |  | Sum | 652 | 1.0001 | 1.00008 | 0.1752 |
|  | |  | |  | |  | |  | |  |  |  |  |  |  |
| Chi-square chi2(8) = 70.75 | | | | | | | | | |  | Chi-square chi2(8)=72.69 | | | | |
| Prob>chi2 = 0.00 | | | | | | | | | |  | Prob>chi2 = 0.00 | | | | |
|  | |  | |  | |  | |  | |  |  |  |  |  |  |
| **Rudnick & Milton-ACH Model** | | | | | | | | | |  | **Applied SEIR Model** | | | | |
|  | |  | | **Observed** | | **Fitted** | |  | |  |  |  | **Observed** | **Fitted** |  |
| **# PTB cases** | | **Abs. Freq.** | | **Rel. Freq.** | | **Rel. Freq.** | | **Abs. Dif.** | |  | **# PTB cases** | **Abs. Freq.** | **Rel. Freq.** | **Rel. Freq.** | **Abs. Dif.** |
| 0 | | 589 | | 0.9034 | | 0.9908 | | 0.0874 | |  | 0 | 589 | 0.9034 | 0.9909 | 0.0875 |
| 1 | | 46 | | 0.0706 | | 0.0091 | | 0.0614 | |  | 1 | 46 | 0.0706 | 0.009 | 0.0615 |
| 2 | | 15 | | 0.023 | | 8.40E−05 | | 0.0229 | |  | 2 | 15 | 0.023 | 7.80E−05 | 0.0229 |
| 3 | | 2 | | 0.0031 | | 9.20E−07 | | 0.0031 | |  | 3 | 2 | 0.0031 | 7.20E−07 | 0.0031 |
| 4 | | 0 | | 0 | | 1.20E−08 | | 1.20E−08 | |  | 4 | 0 | 0 | 7.30E−09 | 7.30E−09 |
| 5 | | 0 | | 0 | | 1.90E−10 | | 1.90E−10 | |  | 5 | 0 | 0 | 7.70E−11 | 7.70E−11 |
| 6 | | 0 | | 0 | | 3.30E−12 | | 3.30E−12 | |  | 6 | 0 | 0 | 8.40E−13 | 8.40E−13 |
| 7 | | 0 | | 0 | | 6.60E−14 | | 6.60E−14 | |  | 7 | 0 | 0 | 9.20E−15 | 9.20E−15 |
| 8 or more | | 0 | | 0 | | 1.30E−08 | | 1.30E−08 | |  | 8 or more | 0 | 0 | 1.30E−09 | 1.30E−09 |
| Sum | | 652 | | 1.0001 | | 0.99998 | | 0.1748 | |  | Sum | 652 | 1.0001 | 0.99998 | 0.175 |
|  | |  | |  | |  | |  | |  |  |  |  |  |  |
| Chi-square chi2(8) = 152.54 | | | | | | | | | |  | Chi-square chi2(8) = 187.27 | | | | |
| Prob>chi2 = 0.00 | | | | | | | | | |  | Prob>chi2 = 0.00 | | | | |
|  | |  | |  | |  | |  | |  |  |  |  |  |  |
|  |  | |  | |  | |  | |  |  |  |  |  |  |  |
| **Rudnick & Milton-L/s/p Model** | | | | | | | | | |  | **List of Abbreviations** | | | | |
|  | |  | | **Observed** | | **Fitted** | |  | |  | #PTB cases = Count of pulmonary TB cases | | | | |
| **# PTB cases** | | **Abs. Freq.** | | **Rel. Freq.** | | **Rel. Freq.** | | **Abs. Dif.** | |  | Abs. Freq. = Absolute frequency | | | | |
|  | |  | |  | |  | |  | |  | Rel. Freq. = Relative frequency | | | | |
| 0 | | 589 | | 0.9034 | | 0.9902 | | 0.0868 | |  | Abs. Dif. = Absolute difference | | | | |
| 1 | | 46 | | 0.0706 | | 0.0098 | | 0.0608 | |  | Chi2(8) = Chi squared goodness-of-fit test with 8  degrees of freedom | | | | |
| 2 | | 15 | | 0.023 | | 9.00E−05 | | 0.0229 | |  |  |  |  |  |  |
| 3 | | 2 | | 0.0031 | | 8.70E−07 | | 0.0031 | |  |  |  |  |  |  |
| 4 | | 0 | | 0 | | 9.40E−09 | | 9.40E−09 | |  |  |  |  |  |  |
| 5 | | 0 | | 0 | | 1.20E−10 | | 1.20E−10 | |  |  |  |  |  |  |
| 6 | | 0 | | 0 | | 1.70E−12 | | 1.70E−12 | |  |  |  |  |  |  |
| 7 | | 0 | | 0 | | 3.00E−14 | | 3.00E−14 | |  |  |  |  |  |  |
| 8 or more | | 0 | | 0 | | 1.10E−08 | | 1.10E−08 | |  |  |  |  |  |  |
| Sum | | 652 | | 1.0001 | | 1.00009 | | 0.1736 | |  |  |  |  |  |  |
|  | |  | |  | |  | |  | |  |  |  |  |  |  |
| Chi-square chi2(8) = 305.01 | | | | | | | | | |  |  |  |  |  |  |
| Prob>chi2 = 0.00 | | | | | | | | | |  |  |  |  |  |  |
