# Supplementary material for: Performance of five dynamic models in predicting tuberculosis incidence in three prisons in Thailand
Source: PLoS One. 2025 Jan 24;20(1):e0318089. doi: 10.1371/journal.pone.0318089 (PMC11761622; doi:10.1371/journal.pone.0318089)
Supplement: S3 Table — (DOCX) [file pone.0318089.s004.docx]

**S3 Table** Fitted models of the negative binomial regression (NBRG) for the five dynamic models in the sensitivity analysis (n=333)

| **Prediction Model** | **Range of observed probability (%) (x1)** | **Constant (b0)** | **Beta (b1)** | **SD** | **IRR** | **(95%CI)** |
| --- | --- | --- | --- | --- | --- | --- |
| Wells–Riley | 0 to 77.6 | −4.945 | 0.024 | 0.007 | 1.024 | (1.011, 1.038) |
| Rudnick&Milton(ACH) | 0 to 53.3 | −5.136 | 0.036 | 0.009 | 1.037 | (1.019, 1.055) |
| Rudnick&Milton(L/s/p) | 0.1 to 35.5 | −4.833 | 0.036 | 0.013 | 1.036 | (1.010, 1.065) |
| Issarow et al. | 0 to 88.4 | −5.110 | 0.035 | 0.009 | 1.035 | (1.017, 1.054) |
| Applied SEIR | 15.6 to 57.0 | −5.517 | 0.025 | 0.008 | 1.025 | (1.009, 1.042) |

CI=Confidence interval, IRR=Incidence rate ratio, SD=standard deviation

Equation used for prediction: Cj = exp[ln(Ej) + b0 + b1x1j], where C=expected number of PTB cases, j=j^th^ cell of the prison, E=the exposure of person-years of observation, b0=constant or intercept, b1=beta coefficient or slope, x1= TB transmission probability estimated by the specified dynamic model, exp=exponential function, ln=natural logarithm.
