# Supplementary material for: Performance of five dynamic models in predicting tuberculosis incidence in three prisons in Thailand
Source: PLoS One. 2025 Jan 24;20(1):e0318089. doi: 10.1371/journal.pone.0318089 (PMC11761622; doi:10.1371/journal.pone.0318089)
Supplement: S4 Table — (DOCX) [file pone.0318089.s005.docx]

**S4 Table** In-sample goodness-of-fit and discrimination test results of five dynamic models in the sensitivity analysis (in-sample, n=333)

| **Prediction Model** | **Goodness-of-fit test results** | | | | **Discrimination tests** | | **10-fold cross-validation** |
| --- | --- | --- | --- | --- | --- | --- | --- |
|  | **Chi-square** | **RMSE** | **MAE** | **Bias** | **AIC** | **BIC** | **Average log likelihoods** |
| Wells–Riley | 69.60 | 0.377 | 0.461 | -0.009 | 463.898 | 475.322 | -237.350 |
| Rudnick&Milton(ACH) | 69.56 | 0.385 | 0.464 | -0.013 | 459.417 | 470.842 | -235.353 |
| Rudnick&Milton(L/s/p) | 69.67 | 0.388 | 0.473 | -0.004 | 468.935 | 480.360 | -241.813 |
| Issarow et al. | 70.56 | 0.382 | 0.666 | 0.223 | 458.568 | 469.992 | -318.560 |
| Applied SEIR | 70.27 | 0.383 | 0.473 | 0.018 | 466.392 | 477.817 | -240.251 |

RMSE=Root Mean Square Error, MAE=Mean absolute error, AIC=Akaike information criterion, BIC=Bayesian information criterion
