# Supplementary material for: Performance of five dynamic models in predicting tuberculosis incidence in three prisons in Thailand
Source: PLoS One. 2025 Jan 24;20(1):e0318089. doi: 10.1371/journal.pone.0318089 (PMC11761622; doi:10.1371/journal.pone.0318089)
Supplement: S5 Table — (DOCX) [file pone.0318089.s006.docx]

**S5 Table** Comparison of accuracy and bias metrics from external validation in the sensitivity analysis (out-of-sample, n=652)

| **Prediction Model** | **Range of observed probability (%) (x1′)** | **RMSE** | **MAE** | **Bias** | **Min predicted value** | **Max predicted value** | **Calibration** | |
| --- | --- | --- | --- | --- | --- | --- | --- | --- |
|  |  |  |  |  |  |  | **slope** | **intercept** |
| Wells–Riley | 0.4 to 71.8 | 0.377 | 0.184 | −0.002 | 0.008 | 1.532 | 0.998 | 0.002 |
| Rudnick&Milton(ACH) | 0.6 to 53.3 | 0.385 | 0.179 | -0.026 | 0.007 | 0.615 | 1.409 | −0.015 |
| Rudnick&Milton(L/s/p) | 0.1 to 35.5 | 0.388 | 0.185 | -0.022 | 0.009 | 0.555 | 1.518 | -0.033 |
| Issarow et al. | 0.1 to 88.4 | 0.383 | 0.172 | -0.022 | 0.007 | 2.036 | 0.939 | 0.028 |
| Applied SEIR | 15.6 to 57.0 | 0.383 | 0.182 | -0.021 | 0.007 | 0.474 | 1.435 | -0.024 |

RMSE=Root Mean Square Error, MAE=Mean absolute error

x1′= TB transmission probability at baseline of the out-of-sample which was estimated by the specified dynamic model
