# Supplementary material for: Performance of five dynamic models in predicting tuberculosis incidence in three prisons in Thailand
Source: PLoS One. 2025 Jan 24;20(1):e0318089. doi: 10.1371/journal.pone.0318089 (PMC11761622; doi:10.1371/journal.pone.0318089)
Supplement: S6 Table — (DOCX) [file pone.0318089.s007.docx]

**S6 Table** TB incidence rate ratio (IRR) from the NBRM demonstrating effect of each variable in the model on PTB incidence rate predicted by five prediction models (*n* = 985)

| **Cell characteristics** | **Crude IRR** | | **The Wells–Riley model** | | | | **The Rudnick & Milton-proposed model (ACH)** | | | **The Rudnick & Milton-proposed model (L/s/p)** | | **The Applied SEIR TB transmission model** | | **Issarow *et al*.’s model** | | |
| --- | --- | --- | --- | --- | --- | --- | --- | --- | --- | --- | --- | --- | --- | --- | --- | --- |
|  | **IRR** | **(95% CI)** | **IRR** | **(95% CI)** | | | **IRR** | **(95% CI)** | | **IRR** | **(95% CI)** | **IRR** | **(95% CI)** | **IRR** | **(95% CI)** | |
| **Architectural characteristics** |  | | | | | | | | | | | | |  | |  |
| **Cell volume (m^2^)** |  |  |  |  | | |  |  | |  |  |  |  |  | |  |
| <30.00 | Ref. |  |  |  | | | Ref. |  | | Ref. |  |  |  |  | |  |
| 30.01–60.00 | 1.19 | (0.59, 2.38) |  |  | | | 1.65 | (0.21, 12.70) | | 3.08 | (0.40, 23.99) |  |  |  | |  |
| 60.01–120.00 | 1.46 | (0.85, 2.52) |  |  | | | 1.50 | (0.18, 12.92) | | 3.06 | (0.36, 26.04) |  |  |  | |  |
| > 120.00 | 1.74 | (0.92, 3.29) |  |  | | | 1.30 | (0.15, 11.29) | | 1.86 | (0.21, 16.16) |  |  |  | |  |
| **Ventilation rate (ACH)** |  |  |  |  | | |  |  | |  |  |  |  |  | |  |
| <12 | Ref. |  | Ref. |  | | |  |  | |  |  | Ref. |  | Ref. | |  |
| 12.01–30.00 | 0.57 | (0.38, 0.87) | 0.76 | (0.50, 1.16) | | | 0.74 | (0.48, 1.16) | |  |  | 0.76 | (0.50, 1.17) | 0.72 | | (0.48, 1.08) |
| 30.01–45.00 | 0.23 | (0.13, 0.42) | 0.35 | (0.19, 0.65) | | | 0.28 | (0.14, 0.53) | |  |  | 0.29 | (0.16, 0.54) | 0.26 | | (0.14, 0.49) |
| 45.01–60.00 | 0.21 | (0.10, 0.45) | 0.32 | (0.15, 0.70) | | | 0.25 | (0.11, 0.58) | |  |  | 0.25 | (0.11, 0.56) | 0.24 | | (0.11, 0.55) |
| > 60.00 | 0.10 | (0.05, 0.24) | 0.11 | (0.04, 0.26) | | | 0.13 | (0.05, 0.37) | |  |  | 0.09 | (0.03, 0.22) | 0.12 | | (0.05, 0.29) |
| **Absolute ventilation rate (L/s/p) †** |  |  |  |  | | |  |  | |  |  |  |  |  | |  |
| <15 | Ref. |  |  |  | | |  |  | | Ref. |  |  |  |  | |  |
| 16–30 | 1.06 | (0.63, 1.80) |  |  | | |  |  | | 0.95 | (0.54, 1.67) |  |  |  | |  |
| 31–60 | 0.62 | (0.34, 1.13) |  |  | | |  |  | | 0.43 | (0.22, 0.85) |  |  |  | |  |
| > 60 | 0.52 | (0.19, 1.42) |  |  | | |  |  | | 0.56 | (0.18, 1.79) |  |  |  | |  |
| **Demographic characteristics of cell inmates** |  | | | | | | | | | | | | |  | |  |
| **Number of inmates in the cell†** |  |  |  |  | | |  |  | |  |  |  |  |  | |  |
| <5 | Ref. |  | Ref.* |  | | | Ref.* |  | | Ref.* |  | Ref. |  | Ref. | |  |
| 6–10 | 0.90 | (0.42, 1.94) | 0.50 | (0.14, 1.79) | | | 0.52 | (0.12, 2.19) | | 0.45 | (0.10, 2.00) | 0.72 | (0.18, 2.84) | 0.51 | | (0.12, 2.17) |
| 11–15 | 0.60 | (0.25, 1.46) | 0.22 | (0.05, 0.92) | | | 0.25 | (0.05, 1.38) | | 0.19 | (0.03, 1.10) | 0.41 | (0.07, 2.40) | 0.25 | | (0.05, 1.33) |
| 16–20 | 1.33 | (0.40, 4.47) | 0.74 | (0.15, 3.72) | | | 1.06 | (0.11, 10.38) | | 0.77 | (0.08, 7.46) | 1.02 | (0.14, 7.63) | 0.74 | | (0.12, 4.56) |
| 21–25 | 1.09 | (0.59, 2.03) | 0.49 | (0.14, 1.75) | | | 0.53 | (0.07, 4.19) | | 0.50 | (0.06, 4.04) | 0.89 | (0.13, 6.29) | 0.36 | | (0.07, 1.73) |
| > 25 | 1.37 | (0.78, 2.43) | 0.39 | (0.11, 1.34) | | | 0.50 | (0.06, 4.26) | | 0.39 | (0.05, 3.27) | 0.46 | (0.07, 2.88) | 0.33 | | (0.07, 1.60) |
| **Area per person (m^3^/person)** |  |  |  |  | | |  |  | |  |  |  |  |  | |  |
| <3 | Ref.* |  | Ref.* |  | | | Ref.* |  | | Ref.* |  | Ref. |  | Ref.* | |  |
| 3.01–4.00 | 0.87 | (0.38, 1.97) | 1.03 | (0.43, 2.48) | | | 4.81 | (1.01, 22.96) | | 1.45 | (0.57, 3.69) | 1.07 | (0.45, 2.55) | 3.43 | | (0.73, 16.16) |
| 4.01–5.00 | 0.72 | (0.46, 1.12) | 0.62 | (0.31, 1.26) | | | 3.15 | (0.56, 17.66) | | 1.16 | (0.36, 3.80) | 0.41 | (0.17, 0.98) | 1.98 | | (0.32, 12.28) |
| 5.01–6.00 | 0.87 | (0.55, 1.38) | 0.56 | (0.26, 1.21) | | | 5.16 | (0.81, 32.84) | | 1.71 | (0.43, 6.83) | 0.44 | (0.20, 1.00) | 2.58 | | (0.41, 16.37) |
| > 6.00 | 1.16 | (0.65, 2.07) | 0.93 | (0.40, 1.21) | | | 7.68 | (1.23, 48.09) | | 2.58 | (0.67, 9.97) | 0.65 | (0.26, 1.61) | 4.00 | | (0.60, 26.47) |
| **Inmate turnover rate (%/year)** |  |  |  |  | | |  |  | |  |  |  |  |  | |  |
| <2 | Ref. |  |  |  | | |  |  | |  |  | Ref. |  |  | |  |
| 2.01–5.00 | 0.83 | (0.36, 1.90) |  |  | | |  |  | |  |  | 0.62 | (0.13, 2.85) |  | |  |
| 5.01–10.00 | 1.04 | (0.58, 1.86) |  |  | | |  |  | |  |  | 0.47 | (0.09, 2.42) |  | |  |
| > 10 | 1.67 | (0.99, 2.82) |  |  | | |  |  | |  |  | 0.61 | (0.09, 4.14) |  | |  |
| **Time-to-TB diagnosis in the cell (days)** |  |  |  |  | | |  |  | |  |  |  |  |  | |  |
| <100 | Ref. |  | Ref. |  | | | Ref. |  | | Ref. |  | Ref.* |  | Ref. | |  |
| 101–140 | 1.10 | (0.55, 2.19) | 0.41 | (0.13, 1.30) | | | 0.17 | (0.00, 6.54) | | 0.01 | (0.00, 0.44) | 1.09 | (0.22, 5.45) | 0.74 | | (0.03, 17.62) |
| 141–180 | 1.05 | (0.53, 2.06) | 0.45 | (0.13, 1.52) | | | 0.08 | (0.00, 14.04) | | 0.00 | (0.00, 0.32) | 1.22 | (0.17, 8.55) | 0.33 | | (0.00, 43.26) |
| > 180 | 1.07 | (0.55, 2.07) | 0.42 | (0.14, 1.23) | | | 0.16 | (0.00, 5.35) | | 0.01 | (0.00, 0.43) | 1.03 | (0.20, 5.37) | 0.60 | | (0.03, 13.53) |
| **Number of overall TB cases in the cell** |  |  |  |  | | |  |  | |  |  |  |  |  | |  |
| 0 | Ref. |  | Ref. |  | | | Ref. |  | | Ref. |  | Ref. |  | Ref. | |  |
| 1 | 2.05 | (1.30, 3.23) | 2.02 | (1.29, 3.14) | | | 4.77 | (2.12, 10.73) | | 5.35 | (2.42, 11.81) | 1.89 | (1.20, 2.97) | 5.23 | | (2.30, 11.88) |
| > 1 | 4.60 | (3.04, 6.95) | 3.45 | (2.33, 5.12) | | | 4.79 | (3.02, 7.59) | | 6.17 | (3.89, 9.79) | 3.46 | (2.31, 5.18) | 5.07 | | (3.15, 8.15) |
| **Number of TB cases in the cell by smear status†** |  |  |  |  | | |  |  | |  |  |  |  |  | |  |
| No | Ref. |  |  |  | | | Ref. |  | | Ref. |  |  |  | Ref. | |  |
| Yes, but smear-negative | 1.96 | (1.08, 3.56) |  | |  | 0.38 | | | (0.16, 0.91) | 0.36 | (0.15, 0.84) |  |  | 0.35 | | (0.15, 0.83) |
| Yes, and smear-positive | 2.19 | (1.40, 3.42) |  | |  | | 0.38 | | (0.18, 0.80) | 0.31 | (0.15, 0.84) |  |  | 0.35 | | (0.16, 0.74) |
| Yes, and both smear-negative and smear-positive | 6.30 | (4.04, 9.84) |  | |  | |  | |  |  |  |  |  |  | |  |
| **Prevalence of TB cases in the zone ¥** |  |  |  |  | | |  |  | |  |  |  |  |  | |  |
| **Smear-negative TB cases** |  |  |  |  | | |  |  | |  |  |  |  |  | |  |
| <0.30 | Ref. |  |  |  | | | Ref. |  | | Ref. |  |  |  | Ref. | |  |
| 0.30–0.50 | 0.61 | (0.38, 0.98) |  |  | | | 0.08 | (0.01, 0.96) | | 0.04 | (0.00, 0.43) |  |  | 0.08 | | (0.01, 0.92) |
| 0.51–1.00 | 0.88 | (0.52, 1.51) |  |  | | | 2.15 | (0.20, 22.87) | | 1.19 | (0.12, 12.00) |  |  | 0.70 | | (0.14, 3.63) |
| > 1.00 | 1.42 | (0.95, 2.10) |  |  | | | 1.50 | (0.10, 21.87) | | 1.41 | (0.11, 18.66) |  |  | 0.45 | | (0.06, 3.32) |
| **Smear-positive TB cases** |  |  |  |  | | |  |  | |  |  |  |  |  | |  |
| <0.20 | Ref. |  |  |  | | | Ref. |  | | Ref. |  |  |  | Ref. | |  |
| 0.20–0.50 | 1.83 | (0.93, 3.60) |  |  | | | 0.54 | (0.04, 6.76) | | 1.11 | (0.01, 1.30) |  |  | 0.51 | | (0.04, 6.11) |
| 0.51–0.80 | 0.94 | (0.44, 2.00) |  |  | | | 0.08 | (0.00, 6.45) | | 0.01 | (0.00, 0.58) |  |  | 0.07 | | (0.00, 6.11) |
| > 0.80 | 2.66 | (1.45, 4.86) |  |  | | | 0.28 | (0.02, 3.49) | | 0.07 | (0.01, 0.91) |  |  | 0.25 | | (0.02, 3.40) |
| **Smear-negative and smear-positive TB cases** |  |  |  |  | | |  |  | |  |  |  |  |  | |  |
| <0.50 | Ref. |  | Ref. |  | | | Ref. |  | | Ref. |  | Ref. |  | Ref. | |  |
| 0.50–1.00 | 0.95 | (0.52, 1.73) | 1.53 | (0.55, 4.25) | | | 1.82 | (0.15, 22.89) | | 9.15 | (0.81, 103.12) | 1.64 | (0.70, 3.83) | 1.85 | | (0.16, 21.96) |
| 1.01–1.70 | 1.54 | (0.82, 2.91) | 1.65 | (0.55, 4.91) | | | 1.23 | (0.09, 17.10) | | 12.73 | (1.08,150.86) | 1.15 | (0.50, 2.62) | 1.23 | | (0.09, 16.03) |
| > 1.70 | 1.98 | (1.12, 3.48) | 1.66 | (0.63, 4.35) | | | 0.24 | (0.02, 3.66) | | 0.77 | (0.05, 10.86) | 2.01 | (0.79, 5.13) | 0.75 | | (0.08, 7.36) |

^* The parameter was not adjusted in the multiple linear regression model.^

^¥^ The prevalence of infectious patients in the zone was obtained by dividing the number of TB cases in the zone by number of inmates in the zone. This data was recorded as a percentage per 6 months during July–December 2020 for Prisons A and B, and during October 2020–March 2021 for Prison C.
