# Supplementary material for: Performance of five dynamic models in predicting tuberculosis incidence in three prisons in Thailand
Source: PLoS One. 2025 Jan 24;20(1):e0318089. doi: 10.1371/journal.pone.0318089 (PMC11761622; doi:10.1371/journal.pone.0318089)
Supplement: Attachment — Submitted filename: 19_Aug_24_Responses_to_Editor_pp2.docx [file pone.0318089.s008.docx]

**Responses to Editor’s and Reviewers’ comments**

Dear Editor and Reviewers,

Thank you very much for your valuable comments. This, however, resulted in the extensive revision of our manuscript for assuring an adequate addressing to your critical comments, with markedly differ from the original manuscript. This may require you to read the whole manuscript again. The responses are as follows.

**The Academic Editor:**

| **Comment** | **Response** |
| --- | --- |
| Reviewers have raised major concerns about the study design, presentation, and results. Please, thoroughly address these before your manuscript can be given further consideation. | Thanks for your valuable comment. The study design, presentation, results, and conclusion have been revised in our primary manuscript. |

**Reviewer 1:**

| **Comment** | **Response** |
| --- | --- |
| Study design was not clearly mentioned in the methodology | We have revised the statement about the study design by emphasizing that: (a) each of the 985 cells constitutes a unit of analysis, not individual inmates; (b) baseline TB transmission probability is the exposure or independent variable; and (c) new PTB cases and incidence rate during the follow-up period are the dependent variable or outcome of interest (page 5, lines 172–174). |
| The statement of outcome is not clear | We provide more detail about PTB outcome, as shown on Page 5, Lines 214-236. |
| Even if the identity of participants are not collected | We have emphasized that the unit of analysis is the cell, not the individual inmate (page 5, line 174).  We have also addressed this as a limitation of our study, as shown on page 16, lines 541–543. |
| Consent is required you are assessing the PTB status | We have provided information that we assessed the PTB status of the inmates indirectly via secondary data, not with the infected inmates (page 6, lines 226–228).  We, however, sought the consent of the superintendent of each prison (page 5, lines 181–187). |
| There is no mention about how the prisoners were tested and who done the medical investigations | We have provided the relevant details on page 6, lines 214–223. |
| The presentation of results in the table could have been better | Because of the statistical analysis’s elaborate revision, all tables have been modified (Pages 9, 10, 11, 12 and S1 Table) |
| Rationale is not clear & the objectives are not connected to the rationale | We have responded to this comment by revising the content in the “Introduction,” as shown on page 2 - 3, lines 61–68, and 71–76. |
| From the stated objective how do you assess the predictive validity of the models under consideration | We have employed less promising terms such as “assess the performance of the dynamic models in predicting future PTB incidence,” as shown in the title (page 1, line 2), and the objective (page3, lines 72–76).  We have revised our statistical analysis by using a four-step procedure including model specification tests, in-sample model fitting, internal validation, and external validation (pages 6–8, lines 238–304). |
| The Five models under consideration are not mentioned clearly in the introduction or methodology | We have provided a brief description of these models on pages 3–5, lines 79–168. |
| Terminologies were suddenly introduced without the rationale of using the terminologies. e.g. deciles of the probability? why is it occurring unanticipatedly | Due to the extensive revision of “the statistical analysis” (pages 6–8, lines 238–304), such terminologies are no longer present in the text. |
| There were no strong literature for the stated objectives | We have responded to this comment by revising the content in the “Introduction,” as shown on page 2 - 3, lines 61–68, and 71–76. |
| Lot of Statistical analysis was performed which are not contributing in meeting the objectives | We have revised our statistical analysis using a four-step procedure including model specification tests, in-sample model fitting, internal validation, and external validation (pages 6–8, lines 238–304).  We have also extensively revised the results (pages 8–13, lines 306-436), the discussion (pages 14–15, lines 443-447, and 477-530), the conclusion (pages16, lines554-563), and the abstract (page 2, lines 20-40). |

**Reviewer 2:**

| **Comment** | **Response** |
| --- | --- |
| This is a research more confirming existing knowledge rather than creating new one. | To avoid repetition with the existing knowledge, we have deleted the phrase “Objective (b) to determine the association between each parameter of the five dynamic models and the occurrence of PTB in Objective (a)” from our manuscript.  We focused only on examining "the performance of the dynamic models in predicting future PTB incidence in prison setting" because existing knowledge on this topic is based solely on simulation research; thus, we reexamined it in a real-world situation. |
| It is a good practice with scientific value to challenge and re-confirm scientific theories. | Our finding challenges the presumption that progressively improved prediction accuracy of the latter models compared to the former models by providing the finding that the original model performed the best in the real-world setting, as shown in the discussion section (pages 14 - 15, lines 443–530), conclusion (pages16, lines554-563), abstract (page 2, lines 20-40) |
| It was said the method was a prospective cohort. However, description of lines 169-177 left me an impression of a case-control study. | Due to the extensive revision of “the statistical analysis” (pages 6–8, lines 238–304), such description no longer exists in the manuscript text.  We have revised the table heading from “the comparison of cells with versus without new PRB cases’ to the comparison of the in-sample versus the out-of-sample’ (for the internal and external validations respectively) (pages 9, lines 349-357).  We have also revised the content in the “Results” section on page 8, lines 312–319, to avoid conveying the impression of a case-control study. |
| A condition for cohort is to assure the equivalence of groups at the beginning except the exposure variable as it began with grouping the result into two (with and without new cases) and then investigated into the risk factors. | We are unable to perform what you indicated since our exposure of interest (i.e., the baseline TB transmission probability) is a continuous rather than a dichotomous variable.  We regarded baseline differences among cells in terms of their architectural, demographic, and health history characteristics as unidentified risk factors and potential confounders that we were unable to consider. We have provided a statement about these limitations of our study on page 15, lines 590–521. |
| The authors did not detail how the 6 categories were defined and how to justify they were equivalent (in addition to “sufficient number of samples in each category”) and only differ by one variable between each other. | Due to the extensive revision of “the statistical analysis” (pages 6–8, lines 238–304), such categorization of the variables no longer exists in the manuscript text. |
| - Line 154-159: how did you manage any conflict between the survey/interview and documents / computer databases? | We have revised the statement to make it more clear that we “ascertained PTB cases via the secondary data of the central administrative and medical facilities in the prisons, then sought further information about the cell locations of the PTB inmates (where they live) by the surveys and interviews with zonal staff and inmates who are prison health volunteers” (page 6, lines 226–230). |
| Figure 2: I guess the y-axis should be “observed” instead of “predicted”? | Due to the extensive revision of “the statistical analysis” (pages 6–8, lines 238–304) and “Results” sections (pages 8- 13), such mistake in the presentation no longer exists. |
| Figure 2: how to explain the hike at decile = 2 for three models? | Due to the extensive revision of “the statistical analysis” (pages 6–8, lines 238–304) and “Results” sections (pages 8- 13), such findings are no longer present in the text. |
