# Supplementary material for: Performance of five dynamic models in predicting tuberculosis incidence in three prisons in Thailand
Source: PLoS One. 2025 Jan 24;20(1):e0318089. doi: 10.1371/journal.pone.0318089 (PMC11761622; doi:10.1371/journal.pone.0318089)
Supplement: Attachment — Submitted filename: 30 DEC 24 Response_to_Reviewers_Comments_R2.docx [file pone.0318089.s009.docx]

**Responses to Editor’s and Reviewers’ comments**

PONE-D-24-03144R2

Dear Editor and Reviewers,

Thank you for your constructive comments that both stimulated and guided us in improving our manuscript, which had also enhanced our research knowledge and skill particularly in statistical analytical aspect. The responses are as follows.

**The Editor:**

| **Comment** | **Response** |
| --- | --- |
| Thank you very much for addressing the reviewers comments. However, new concerns have been raised by the reviewers which need to be addressed before the manuscript can be given further consideration. | Thanks for your valuable comment. All new concerns have been revised in our primary manuscript. |

**Reviewer #3:**

Thank you very much sir/madam for your favorable opinion.

**Reviewer #4:**

**Minor Comments**

| **Comment** | **Response** | |
| --- | --- | --- |
| The readability of the paper can be improved - it felt like the paper had not been proof-read before submission. | Your comments are greatly appreciated. We had meticulously proofread the manuscript before submission. | |
| The presentation of the models I believe should be in the methods section rather than the introduction section. | We followed your recommendation, as illustrated on lines 79–167 of pages 4–7. | |
| Variables explained in the text in relation to the models' equations are not typed in 'math' mode and thus make it hard to follow. | Thank you for your feedback. In accordance with your suggestion, the models’ equations have been modified to be in the “math” mode, as demonstrated on page 4, lines 89, page 5, line 110 and on page 6, line 130. | |
| Furthermore, some variables are repeated (given most of the models are extensions of others surely there is a better way to explain the parameters without repetition). | Although certain variables are repeated or represented using the same symbols, there are differences in the details and methods employed to calculate the probability of tuberculosis transmission in each model. Our approach is consistent with the definitions outlined in the original research articles. For instance, the variable ventilation rate in Rudnick & Milton’s modified model can be denoted in two distinct ways: air changes per hour (ACH) or liters per second per person. Conversely, other models in this study exclusively use ACH. Similarly, the unit for the variable t in the classic Wells–Riley model is expressed in months, whereas the unit for the variable t is in days in Rudnick & Milton’s modified model. Furthermore, the variable t is not included in the SEIR model.  Therefore, we continue to present the detailed specifications of each model to ensure that readers or researchers intending to utilize the information from this study can do so without any difficulty or confusion. The dataset files, which are accessible from the Figshare repository (DOI: 10.6084/m9.figshare.26787874), contain the calculation methodologies. However, we have removed specific variables that were previously described in the Wells–Riley model. | |
| Line 114 - parameter \theta is mentioned but it is not actually present in the equation above. | In accordance with your comment, we have made the necessary revisions to the content on page 5, line 110. | |
| Line 117 - 'per person' mentioned twice. | As per your recommendation, we have made the necessary revisions to line 117 on page 6. | |
| There is generally an inconsistency in how units are presented (e.g. per hour vs hr^{-1}) | We have replaced the terms “hr” with “h” and “hr^−1^” with “/hour,” as shown on Page 6, lines 135, 137 and 138 respectively. | |
| Line 132 - first define \beta and \mu and then define (\beta - \mu). | We implemented your recommendation, as illustrated on lines 132–135 of page 6. |  |
| Line 139 - parameter p defined twice? | The parameter p is defined twice, as the second instance of p is a sub-variable used in the calculation of the variable ƒ (the fraction of rebreathed air). | |
| I am a bit confused about the SEIR model and the units used (e.g person/year and person/180 days etc. shouldn't there be consistency?) | The variable number of overall TB cases (infectious) based on actual data has not been utilized in any previous studies, as indicated by the review of related literature. Instead, most studies have relied on assumed values to determine the probability of TB transmission. In contrast, this study monitored TB prevalence and incidence in a real-world setting over a 6-month period (equivalent to 180 days), which is consistent with the standard duration of TB treatment in Thailand prisons. This 180-day or 6-month period was also employed in our calculations of the probability of TB transmission, in accordance with the original studies. | |
| Line 177 - change "5" to "five" to be consistent with the rest of the text. | We have changed "5" to "five” on page 8, line 175, as per your suggestion. | |
| Line 233 - IR already defined in introduction so can use abbreviation. Also in the text incidence rate is sometimes mentioned via the abbreviation and sometimes written out in full. Why introduce the abbreviation if you will not use it consistently? | We have revised the content in line with your comment, as shown on page 10, line 230; page 12, line 271; page16, lines 346 and 347; page 17, line 367, 375 and 380; page 23, line 429, 430 and 440; page 23, line 455. | |
| The prisons are introduced as "Prison A" etc. and then referred to as "prison A" etc. Be consistent with capitalisation. | We have made the necessary modification, as per your recommendation. | |

**Major Comments**

| **Comment** | **Response** |
| --- | --- |
| The in-sample and out-of-sample |  |
| Rationale for selecting the in-sample and out-of-sample | The rationale for selecting the in-sample and out-of-sample is outlined in the “Statistical analysis” section on page 10 and 11, lines 236–243. |
| Other combination of the in-sample and out-of-sample | We conducted the sensitivity analysis by reanalyzing the data after switching the datasets between the in-sample and out-of-sample. We present the statements/contents regarding this activity in:   - The “Statistical analysis” section on page 13, lines 306–310; - The “sensitivity analysis” sub-section of the “Results” section on page 22, lines 417–424, and in Tables S3 to S6; - The “Discussion” section on page 23, lines 434–436. |
| Rationale for selecting count models | The rationale and procedure for selecting the optimal count model are detailed in the “Model specification test” sub-section” of the “Statistical analysis” section on page 11, lines 254–262, and in Table S1.  To execute model fitting, we employed ZIP, ZINB, and NBRG. The results are quite comparable, as illustrated below. Therefore, we incorporated only the NBRG results in the manuscript. |
| **Table** Fitted models for the five dynamic models (n = 652) **Prediction Model** \| **Constant (b0)** \| **Beta (b1)** \| **SD** \| **IRR** \| **(95%CI)** \|; \| \| **NBRG** \| \| \| \|; Wells–Riley \| −5.067 \| 0.030 \| 0.006 \| 1.030 \| (1.018, 1.043) \|; Rudnick & Milton(ACH) \| −5.116 \| 0.051 \| 0.013 \| 1.053 \| (1.025, 1.081) \|; Rudnick & Milton(L/s/p) \| −4.823 \| 0.067 \| 0.028 \| 1.069 \| (1.011, 1.130) \|; Issarow et al. \| −4.875 \| 0.033 \| 0.009 \| 1.033 \| (1.016, 1.051) \|; Applied SEIR \| −5.544 \| 0.031 \| 0.010 \| 1.031 \| (1.011, 1.051) \|; \| \| **ZIP** \| \| \| \|; Wells–Riley \| −4.693 \| 0.028 \| 0.006 \| 1.028 \| (1.017, 1.040) \|; Rudnick & Milton(ACH) \| −4.647 \| 0.050 \| 0.013 \| 1.051 \| (1.024, 1.078) \|; Rudnick & Milton(L/s/p) \| −4.271 \| 0.056 \| 0.026 \| 1.057 \| (1.004, 1.114) \|; Issarow et al. \| −4.450 \| 0.029 \| 0.007 \| 1.030 \| (1.015, 1.045) \|; Applied SEIR \| −5.064 \| 0.031 \| 0.010 \| 1.031 \| (1.011, 1.052) \|; \| \| **ZINB** \| \| \| \|; Wells–Riley \| −4.692 \| 0.028 \| 0.006 \| 1.028 \| (1.016, 1.040) \|; Rudnick & Milton(ACH) \| −4.647 \| 0.050 \| 0.013 \| 1.051 \| (1.024, 1.078) \|; Rudnick & Milton(L/s/p) \| −4.271 \| 0.056 \| 0.027 \| 1.058 \| (1.004, 1.114) \|; Issarow et al. \| −4.450 \| 0.029 \| 0.007 \| 1.030 \| (1.015, 1.045) \|; Applied SEIR \| −5.064 \| 0.031 \| 0.010 \| 1.031 \| (1.011, 1.052) \| CI = Confidence interval, IRR = Incidence rate ratio, SD = standard deviation | |
| Sensitivity analysis to assess robustness or identify critical parameters. | We conducted post-hoc analysis for substantiating our statement that the ventilation rate in ACH is a critical parameter in predicting future PTB incidence (“Discussion” section, page 25, lines 476–479), and the results are presented in Table S6. |

**Main Concerns**

| **Comment** | **Response** |
| --- | --- |
| Comment about ‘further research’ | The “further research” comprised the previously described investigations during sensitivity analysis, which involved switching the datasets between the in-sample and out-of-sample.  Based on the result from the sensitivity analysis, we also incorporated the statement to further support the conclusion regarding the Wells–Riley model’s resilience, as demonstrated in the “Discussion” section on page 26, lines 512–516.  We have however retained the statement regarding “further research” and provided a more detailed explanation, as illustrated in the “Discussion” section on page 26, lines 516–522. |
